# General practitioners and carers: a questionnaire survey of attitudes, awareness of issues, barriers and enablers to provision of services

**DOI:** 10.1186/1471-2296-11-100

**Published:** 2010-12-20

**Authors:** Nan Greenwood, Ann Mackenzie, Ruth Habibi, Christine Atkins, Ray Jones

**Affiliations:** 1 Faculty of Health and Social Care Sciences, St George's University of London and Kingston University, London, UK

## Abstract

**Background:**

Approximately one in ten of the UK population are unpaid carers supporting a family member or friend who could not manage without their help, saving the UK economy an estimated £87 billion. This role is known to sometimes have a negative impact on carers and to require support both informally and from statutory services. General practice is a first point of contact for carers but research investigating general practitioners' (GPs') attitudes towards carers and awareness of issues facing carers is rare. This study therefore aimed to identify GPs' attitudes, awareness of issues, and perceptions of the barriers and enablers to provision of services.

**Methods:**

Using a self-completion questionnaire distributed at a series of workshops, this study investigates GPs' attitudes to carers; awareness and knowledge of carers' issues; services offered in general practice and barriers to supporting carers.

**Results:**

Seventy eight out of a total of 95 GPs (82% response rate) from a variety of areas in England completed the questionnaires. The GPs identified time, resources and lack of knowledge as barriers, but only 9% agreed with the statement that there is little support they can offer carers. However, nine in ten GPs (89%) feel they have insufficient training here and approximately half of them (47%) lack confidence that they are meeting carers' needs. Confidence in identifying carers is also low (45%). Issues that GPs would look out for amongst carers include emotional and physical health problems and financial and isolation difficulties. GPs specifically highlighted educational and isolation issues for young carers. Few services were described that targeted carers.

**Conclusions:**

GPs recognise that they have an important role to play in supporting carers but would like training and support. Further investigation is needed both to determine how best to train and facilitate GPs and general practice teams in their role in supporting carers and to identify what carers need and want from general practice. Identifying carers' leads or carers' champions amongst practice staff is possibly one way forward. Given the proposed greater commissioning role for primary care, greater understanding here is particularly important.

## Background

### Carers

Approximately six million (approximately one in ten) of the population of England and Wales are carers [1] with three in five people becoming carers at some point and numbers are expected to increase with the ageing population and greater numbers of severely disabled people living longer [2].

A carer (also sometimes known as informal carer or caregiver) is defined as:

'.... someone who, without payment, provides help and support to a partner, child, relative, friend or neighbour, who could not manage without their help. This could be due to age, physical or mental illness, addiction or disability. The term carer should not be confused with a care worker, or care assistant, who receives payment for looking after someone.' [3,p9).

Over half of these carers are women (58%) and approximately 175,000 of them are children. Nine hundred thousand carers care for over 50 hours a week [2].

It is now accepted that carers often suffer poorer mental and physical health as a result of their role [4]. For example, amongst carers of people with long-term conditions such as dementia and stroke, negative outcomes include emotional distress, reduced quality of life, burden and stress [5-7]. There is also some evidence that providing unpaid or informal care for a long time is associated with deterioration in both physical and psychological health [8].

It has been estimated that carers save the economy £87 billion annually [9]. The vital role played by these carers is now recognised by the government which has pledged to recognise and support carers [10,11]. The numbers of young carers has also been acknowledged [12].

### General practice and carers

General practice can play a significant role in supporting carers. According to a joint report by the Royal College of General Practitioners (RCGP) and the Princess Royal Trust for Carers (PRTC):

'GPs and their teams are usually the first place that carers have contact with the National Health Service. They are uniquely placed to recognise that someone is, or is about to become, a carer.' [3,p2).

Research has also shown that carers believe that GPs are both well placed to support them and have the power to improve the quality of carers' lives [13]. It might therefore be expected that carers would visit their GPs more than the population as a whole, although the evidence here is inconclusive, possibly in part because of methodological issues [14].

Despite this there is little recent published UK research in this area. A postal survey nearly a decade ago (Simon and Kendrick 2001) [15] found that GPs and primary care teams thought they had a key, pro-active role to play supporting carers but that they lacked time, resources and training.

Research in Australia [16] focussing on GPs' perceptions of carer emotional needs highlighted that although GPs were aware of carers' increased emotional needs, the services they offered were almost exclusively 'practical '(including referring and directing carers to services to ensure the carer had practical assistance). GPs preferred to refer carers to community services although some did offer counselling described as 'informal sharing or 'coffee cup counselling'' (p4).

GP contracts in the UK currently give three points in the Quality and Outcomes Framework (QOF) for the establishment of a system to identify and refer carers to local authorities for assessment of their needs. The Quality and Outcomes Framework (QOF) rewards GP practices for how well they care for their patients and helps fund improvements in the care they deliver. It is based on performance against specified indicators or measures of achievement. Each indicator is worth a maximum number of points and GP practices are rewarded financially according to how many points they achieve. Primary Care Trusts also have a responsibility for establishing clinical governance protocols for the delivery of services by GPs which should include addressing and supporting the role of carers [17].

### Aims

Using a self-completion questionnaire this study aimed to investigate GPs': attitudes to carers; awareness and knowledge of issues facing carers; perceived barriers to supporting carers and services offered to carers.

### The study

The Department of Health (DH) in partnership with the Royal College of General Practitioners (RCGP) commissioned a pilot workshop training programme for GPs and other members of primary care teams across England to learn about carers. The findings reported here were gathered from questionnaires completed by GP participants prior to the workshops and was part of the programme's evaluation.

## Methods

### Setting and method

Six pilot workshops were held in the latter half of 2009 in six general practice Faculties (Bedfordshire and Hertfordshire; North West London; North West England; Vale of Trent; Yorkshire; Wessex). The aim of the workshops was to give participants a better understanding of the problems facing carers and the role that primary care might take in supporting them. Workshops were advertised by the RCGP and invitations were sent out from the relevant general practice Faculty. Where appropriate, workshop participants received credit for their Continuing Professional Development.

All participants were asked to complete the questionnaire when registering for the workshops. Questionnaires were distributed electronically prior to each workshop. Where participants had not completed it earlier, they were again asked to do so on arrival. Although representatives from a range of professions attended the workshops, only responses from the GPs are included here because of the small numbers representing other professional groups.

### Questionnaire

The questionnaire was designed specifically for the study. Topics covered three sections: participant demographics (e.g. age, gender); practice background information (e.g. practice size, staff employed and location); attitudes towards carers; knowledge of issues facing carers and carer specific services offered by participants' practices. Questions were derived from literature focusing on carers and general practice and ideas raised in the 'Supporting Carers: An Action Guide for General Practitioners and Their Teams' (PRTC and RCGP 2008) [3]. Questions were phrased both positively and negatively to reduce the chances of response bias where participants may tend to agree rather than disagree with statements, a behaviour sometimes referred to as 'yeah saying' [18].

The questionnaire included both open-ended and closed questions using Likert scales and 'Yes/No' responses. This method was expected to capture a more complete picture of GP perspectives than using only one approach. The quantitative element provides numerical results whilst the open-ended questions gave the GPs the opportunity to articulate personal perspectives and describe experiences and issues not covered by the Likert scales.

Likert scales measured agreement with statements about, for example, attitudes towards carers and services offered. Participants could choose from 'strongly agree', 'agree', 'neither agree nor disagree', 'disagree', 'strongly disagree' and' don't know'.' For the purposes of the analysis responses were collapsed into 'agree', 'neither agree nor disagree', 'disagree' and 'don't know'. Where appropriate, findings (such as practice details) were subjected to descriptive statistics.

Open-ended questions concerned, for example, social and health issues facing carers and these responses were content analysed.

Questionnaires were piloted with five GPs. The only issues that arose related to the questionnaire layout. However, after the first batch of questionnaires was sent to participants, some objected to being required to specify their age. The questionnaire was therefore altered so participants described their age in ten year categories. Responses from the early questionnaires were retrospectively assigned to these categories.

### Ethics approval

When contacted, the National Research Ethics Service advised that this study did not require ethical review because it was an educational evaluation, not research. It therefore did not require ethical approval. However the proposal was looked at by the local research ethics committee and the study was conducted following ethical principles such as informed consent and respect for confidentiality.

## Results

### Participants, reasons for attending and practice characteristics (Table 1)

**Table 1:** Participants, reasons for attending and practice characteristics

Participants (n = 78)		n (%)
**Gender**	Female	59 (76%)
	Male	19 (24%)

**Age categories**	Under 30	12 (15%)
	30-39	21 (27%)
	40-49	28 (36%)
	50-59	11 (14%)
	60+	3 (4%)
	Missing	3 (4%)

**Reasons for attending**	Increase knowledge	30 (38%)
	Improve services	19 (24%)
	Increase practice knowledge	8 (10%)
	Role	7 (9%)
	Interest	7 (9%)

**Training about carers**	Had received training	29 (37%)
	In practice	12 (15%)
	As a student	9 (12%)
	External organisation	6 (8%)

**Practice details**		

**Location**	Urban	51 (65%)
	Semi-rural	8 (10%)
	Rural	8 (10%)
	Missing	11 (14%)

**List size**	Median	8000
	Min-Max	1700-18000

**Number of GPs**	Median	6
	Min-Max	2-15

**Number of GP trainees**	Median	1
	Min-Max	0-6

Questionnaires were completed by 78 of the 95 GPs attending the workshops (82% response rate). Three-quarters were female (76%) and over half (54%) were aged over 40 years.

Nearly a third (29%) had received previous training in issues in supporting carers. Approximately a third of these said it had been as a student and for a similar proportion training had been during their practice.

GPs were asked an open-ended question about why they attended the workshop and the main reasons they gave were to increase their own (38%) or their practices' (10%) knowledge. For example, they said they hoped to improve their own knowledge in issues facing carers and how best to identify them. Some explicitly mentioned they were attending with the intention of sharing the information gained with others in their practice. A quarter said they hoped to be able to improve the provision of services for carers (24%).

Participants came from varying sized practices. Numbers of GPs working in each practice ranged from two to 15 (median 6). Numbers of GP trainees per practice varied from none to six (median 1).

Patient list sizes ranged from 1700 to 18000 (median 8000). Two thirds of practices were identified as urban (65%), one in ten semi-rural (10%) and one in ten rural (10%).

### The role of general practice in supporting carers, attitudes to carers and issues facing carers

#### Responses to Likert scales (Table 2)

**Table 2:** Summary of responses to Likert scales

	Agree	Neither agree nor disagree	Disagree	Don't know
There is little support that general practice can offer to carers (n = 75)	7(9%)	9 (12%)	58(77%)	1 (1%)

I feel confident that I could identify the carers in my practice (n = 74)	33 (45%)	17 (23%)	21 (28%)	3 (4%)

In general I feel confident that I meet the needs of carers (n = 75)	8 (11%)	27 (36%)	36 (47%)	4(5%)

Supporting carers can be difficult (n = 74)	64 (86%)	4 (5%)	5 (7%)	1 (1%)

If the cared-for person dies, I routinely contact their carer (n = 74)	40(54%)	10 (14%)	17 (23%)	7(9%)

I take an active role in supporting carers (n = 74)	39(53%)	21 (28%)	9 (12%)	5 (7%)

There is little point in referring carers to support services as they are unlikely to use them (n = 75)	1 (1%)	11 (15%)	63 (84%)	0 (0%)

GPs should be pro-active in identifying carers (n = 75)	70 (93%)	3 (4%)	1 (1%)	1(1%)

Carers should be a partner in the health care of their cared-for person (n = 75)	63 (84%)	9(12%)	1 (1%)	2 (3%)

Confidentiality of the cared-for person can be an issue when working with carers (n = 75)	69 (92%)	3 (4%)	3 (4%)	0 (0%)

Carers are often a barrier in managing the healthcare of the cared-for person (n = 75)	7 (9%)	24 (32%)	43 (57%)	1 (1%)

Carers deserve more support from primary care teams (n = 74)	63 (85%)	10 (14%)	1 (1%)	0(0%)

Carers are no more likely to suffer from emotional problems than the public in general (n = 75)	17(23%)	2 (3%)	56 (75%)	0 (0%)

Young carers are more likely to self-harm than other young people (n = 75)	48(64%)	8 (11%)	1 (1%)	18 (24%)

The all-cause mortality rate is increased for carers (n = 75)	48(64%)	10 (13%)	1 (1%)	16 (21%)

Carers frequently have to stop paid employment once they become carers (n = 75)	60 (80%)	8 (11%)	1(1%)	6 (8%)

General practitioners are not trained sufficiently well to support carers (n = 75)	67 (89%)	5 (7%)	3 (4%)	0 (0%)

Carers from some minority ethnic groups are less likely to accept support from primary care (n = 75)	75 (61%)	19 (15%)	5 (4%)	20 (16%)

There are sufficient support services for carers (n = 75)	2 (3%)	7 (9%)	59 (79%)	7 (9%)

Responses to the Likert scales showed that GPs lacked confidence in their role (only 11% said they were confident) and the majority (89%) felt insufficiently trained in supporting carers. Less than half (45%) were confident that they could identify carers in their practice. A few (9%) regarded carers as 'sometimes a barrier in managing the healthcare of the cared-for person' and maintaining confidentiality of the care recipient was recognised as difficult by the majority of these GPs (92%).

Most of these GPs believe that general practice can play a role in supporting carers (77%) and that they should be pro-active in this role (93%). Approximately half said that they already take an active role here (53%). GPs said carers deserve more support (85%) although the majority (86%) agreed that 'supporting carers can be difficult'. Nevertheless more than eight in ten (84%) disagreed with: 'There is little point in referring carers to support services as they are unlikely to use them'. The majority (84%) agreed that carers 'should be a partner in the health care of their cared-for person'.

In terms of knowledge, awareness that carers are more likely to suffer from emotional problems was high. Three-quarters (75%) noted that they were aware of this. Two-thirds of GPs (64%) agreed that carers' all-cause mortality rate is increased whilst a fifth (21%) said they did not know.

### Responses to open-ended questions

#### Health and social issues looked out for amongst carers (Tables 3 and 4)

**Table 3:** GPs' perceptions of social and health issues amongst carers

Responses to: 'Are there any particular social issues amongst carers in general that you would look out for?'		Responses to: 'Are there any particular health issues amongst carers in general that you would look out for?'	
**n = 78**	**n (%)**	**n = 78**	**n (%)**

Finance	30 (38%)	Depression	40 (51%)

Isolation	25 (32%)	Neglect own health	19 (24%)

Mental health	16 (21%)	Physical and chronic health problems	10 (13%)

Support	9 (12%)	Stress	9 (12%)

Time for self	9 (12%)	Anxiety	8 (10%)

Employment	9 (12%)	Mental health	14 (18%)

Fatigue	5 (6%)	Drug and alcohol problems/smoking	6 (8%)

Health	4 (5%)	Fatigue	3 (4%)

Stress	4 (5%)	Social problems	7 (9%)

No response	21 (27%)	No response	15 (19%)

Multiple responses

**Table 4:** GPs' perceptions of problems facing young carers

n = 78	n (%)
Responses to 'Are there any problems that young carers might experience?'	

Education (including missed schooling and poor academic performance)	37 (47%)

Isolation	31 (40%)

Relationships with family/friends	14 (18%)

Depression	9 (12%)

Lack of support	2 (3%)

Emotional problems	8 (10%)

Inappropriate level of responsibility	7 (9%)

Finance	7 (9%)

Reduced life choices/job prospects	6 (8%)

Missed childhood	5 (6%)

No response	9 (12%)

Multiple responses

When asked about social issues amongst carers that they would look out for, approximately a quarter of GPs (27%) provided no answer. Amongst those that responded, the problems most frequently mentioned were financial (38%), isolation (32%) and mental health (21%). Just over one in ten (12%) mentioned each of the following: time for self, employment and support (Table 3).

Mental health issues were most frequently noted as carers' health issues. Some mental health problems were mentioned specifically (depression 51%, stress 12%, anxiety 10%) whilst some participants only referred to 'mental health' in general (18%). Neglect of carers' own health was also highlighted (24%) (Table 3).

When asked specifically about issues for young carers (Table 4) a range of responses were given. Nearly half of the GPs highlighted problems with education including missed schooling and poor academic performance (47%). Isolation was also commonly mentioned (40%). Other concerns included relationship problems with families or friends (18%) and depression (12%).

#### Services for carers and barriers to providing support (Tables 5 and 6)

**Table 5:** Services offered to carers and perceptions of the support GPs think carers would like from general practice

Services offered by GPs		What GPs think carers would like	
Responses to 'Does your practice offer any services specifically for carers?'		Responses to 'What support do you think carers would like from general practice?'	

**n = 78**	**n (%)**	**n = 78**	**n (%)**

Register	5 (6%)	Emotional support	21 (27%)

Flu vaccine	5 (6%)	Signposting/referral to appropriate organisations/agencies	17 (22%)

Carers' pack/leaflets	4 (5%)	Information/advice about services	15 (19%)

Health checks/carer clinics	3 (4%)	Understanding	7 (9%)

Referral to appropriate services	3 (4%)	Respect/recognition	7 (9%)

Notice boards	2 (3%)	Easy access to GP	6 (8%)

Carer support groups	2 (3%)	Information	5 (6%)

In house services from Carer Centres/CAB	2 (3%)	Practical and financial support	5 (6%)

Planning to set up services	2 (3%)	Support	4 (5%)

Signposting	1 (1%)	Information/advice about the care recipient	4 (5%)

Other	5 (6%)	Other	7 (9%)

No response	57 (73%)	No response	14 (18%)

Multiple responses

**Table 6:** Perceived barriers in supporting carers

n = 78	n (%)
Responses to 'Are there any barriers in your work supporting carers?'	

Lack of time	34 (44%)

Financial/resources	18 (23%)

Lack of knowledge	14 (18%)

Difficulty of identifying carers	8 (10%)

Carer attitudes (e.g. self identification, unwilling to accept help)	3 (4%)

GP attitude	3 (4%)

Lack of direct contact with carers	2 (3%)

Confidentiality	2 (3%)

Practice space	2 (3%)

Other	3 (4%)

No response	22 (28%)

Multiple responses

Few participants responded to the open-ended question concerning services offered specifically for carers. Three-quarters (73%) gave no response. Six percent (five GPs) already had a carers' register and the same number offered flu vaccinations, although they did not say if they were specifically offered to carers. Four GPs (5%) had a carers' pack or provided leaflets.

Approximately a quarter of GPs thought carers wanted emotional support (27%) and signposting or referral to relevant agencies (22%). Participants (19%) also thought carers would like information including advice about services (Table 5).

Over a quarter of GPs (28%) did not identify any barriers in supporting carers (Table 6). Those mentioning obstacles highlighted insufficient time (44%) and resources (23%). Lack of knowledge about carers was an issue for nearly one in five participants (18%). Difficulty in identifying carers was raised by one in ten.

## Discussion

Against the backdrop of the likely future increase in numbers of carers because of an ageing population and more people living with improved healthcare so that more people live with disability for longer [2], it is important that general practice and GPs are in a position to support them. This study has demonstrated that although GPs regard general practice as an appropriate place to do this and think they should be pro-active in their role, they frequently lack confidence and training and sometimes knowledge to do it effectively. Key issues highlighted include the identification of carers - some GPs are aware that they are not identifying all carers in their practices and would like to be guided on how best to do this. Few services are currently being offered by general practices specifically for carers despite GPs' belief that carers want their support. The few carers' registers reported here by the GPs is all the more surprising given the financial incentives to have them.

There are several possible reasons for this. GPs may believe that as carers have no specific medical diagnosis and have social not medical problems, supporting them is perhaps only on the periphery of their role [19]. They may also think identification and support of carers may add to an already demanding workload and have concerns that carers may present problems they are unable to help with. However, the GPs in this study agree that carers deserve their support even if insufficient time and resources and concerns about confidentiality remain. Clearly the fact that carers and the person being cared for may not have the same GP or even attend the same practice complicates both identifying carers and may make communication more difficult.

There are noticeable differences between our findings and those of Simon and Kendrick (2001)[15]. Variations in question wording (such as open-ended questions versus checklists in Simon and Kendrick's study) and our sample's attendance at a carers workshop account for some of this, but the differences are still striking. On a positive note, the vast majority of our sample (90%) compared with a quarter in the earlier study said that they should be proactive in supporting carers. Approximately a third of our participants (29%) said they had received some previous training in carers compared with 10% nearly a decade ago. On the other hand, five GPs here (6%) reported keeping a carers' register. In Simon and Kendrick's study, a quarter recorded carer status. Although these activities are not identical, this suggests that identifying carers may remain a low priority. Also nearly half the GPs in Simon and Kendrick's study offered information to carers, compared with a handful here.

There are similarities with our findings and the research from Australia [16]. In both there is an apparent gap between what GPs think carers might like and what they offer them. Despite awareness of carers' emotional needs, GPs in Australia tended to refer carers to practical, rather than emotional support. Similarly here although a quarter of the GPs thought carers would like emotional support, the only service provided that might be directly expected to offer such support was carer support groups available at three practices. GPs here described fewer services for carers than in the Australian study but nearly all those mentioned here were also practical in their approach.

Nearly a decade ago it was suggested that primary care teams could support carers in a number of ways including acknowledging the problems they have and ensuring the general practice team are aware of them, flagging carers' notes so that GPs were aware of their caring role, acknowledging the role they play, treating them as team members and provision of information for the carer relating for example to the condition of the person the carer is looking after and information about benefits and services [19]. The fact that the DH and the RCGPs organised these workshops suggests greater awareness of the importance of carers and the significant role they play but our findings suggest that many of these earlier recommendations still stand. Although most GPs here did agree that carers should be a partner in the care of the recipient of care, little progress seems to have been made with the other recommendations.

However, there is very little recent published literature on the topic and it may be worth speculating why. Perhaps it is a symptom of lack of awareness of the major role played by carers or perhaps an indication of the uncertainly and maybe ambivalence amongst GPs about supporting this group.

Setting up carers registers is an important first step but if GPs are unaware of common problems amongst carers and feel they have insufficient knowledge and time to offer support, raising expectations may be detrimental. A possible approach here is increasing the numbers of carers' leads or champions in primary care teams [20]. Such carers champions are a member of the general practice staff who can recognising the needs and difficulties of carers and be able to offer them information and respond to their enquiries. The impact of this role has not been formally evaluated but they could assume much of the responsibility for supporting and signposting carers.

There remains work to be done. Repeating this survey with GPs who had not opted to attend training on carers would offer a wider perspective although the fact that the participants here had expressed an interest in the area but still lacked some knowledge and awareness of likely problems amongst carers (as suggested by the questions where they failed to answer or were unaware of some facts about carers) can also be seen to further the argument for the value of our findings. Better appreciation of GPs' perspectives and those of other primary care team members is required but research is also needed to identify what support carers need and want from primary care. Possibly adopting qualitative methods would allow a more in-depth understanding of all these perspectives. Research exploring the potential of roles such as carers' champions who might be well-placed to support carers whilst minimising the input from GPs would be beneficial.

### Strengths and limitations

A strength of our study is that we achieved a high response rate for this group of participants [21] and the GP participants came from a range of areas geographically including both rural and urban populations and involved both large and small practices. The response rate was also high. Given the lack of research in the area, in many ways the study is exploratory since it investigated a wide range of issues many of which need further research.

A limitation is that the study participants had mostly chosen to attend a workshop on carers and may have been an atypical group motivated to support carers. It is impossible to know what impact this had on the findings but given the lack of research in the area, this is an important step in understanding GPs perceptions. Importantly these GPs were mostly attending the workshops because they wanted to know more about carers. There were a higher proportion of female GPs and they were slightly younger than might be expected [22] and nearly half said that they had an interest in carers and may therefore have been better informed compared with other GPs, although the current findings suggest they lack some knowledge in the area.

## Conclusions

Without carers costs to the UK economy would increase hugely. GPs recognise general practice has an important role to play in supporting these carers but would appreciate both more training and support. Research is needed both to determine what support carers need and would like from general practice and to evaluate any new roles that are introduced.

## Competing interests

The authors declare that they have no competing interests.

## Authors' contributions

All authors contributed to the study design. NG was primarily responsible for the questionnaire construction. All authors participated in data collection and content analysis of open-ended questions. RH was primarily responsible for the quantitative analysis and coordinated the project. NG drafted the article. All authors read and approved the final manuscript.

## Pre-publication history

The pre-publication history for this paper can be accessed here:

http://www.biomedcentral.com/1471-2296/11/100/prepub
